# Baseline Characterization and Annual Trends of Body Mass Index for a Mega-Biobank Cohort of US Veterans 2011–2017

**Published:** 2018

**Authors:** Xuan-Mai T. Nguyen, Rachel M. Quaden, Rebecca J. Song, Yuk-Lam Ho, Jacqueline Honerlaw, Stacey Whitbourne, Scott L. DuVall, Jennifer Deen, Saiju Pyarajan, Jennifer Moser, Grant D. Huang, Sumitra Muralidhar, John Concato, Philip S. Tsao, Christopher J. O’Donnell, Peter W. F. Wilson, Luc Djousse, David R. Gagnon, J. Michael Gaziano, Kelly Cho

**Affiliations:** 1 Massachusetts Area Veterans Epidemiology Research and Information Center, Veterans Affairs Boston Healthcare System, Boston, MA; 2 Department of Medicine, Division of Aging, Brigham and Women’s Hospital, Boston, MA; 3 Department of Medicine, Harvard Medical School, Boston, MA; 4 Department of Veterans Affairs Salt Lake City Health Care System, Salt Lake City, UT; 5 Department of Internal Medicine, University of Utah School of Medicine, Salt Lake City, UT; 6 Office of Research and Development, Veterans Health Administration, Washington, DC; 7 Veterans Affairs Connecticut Healthcare System, West Haven, CT; 8 Department of Internal Medicine, Yale School of Medicine, New Haven, CT; 9 Veterans Affairs Palo Alto Healthcare System, Palo Alto, CA; 10 Department of Medicine, Stanford University School of Medicine, Stanford, CA; 11 Department of Medicine, Division of Cardiovascular Medicine, Brigham and Women’s Hospital, Boston, MA; 12 Atlanta Veterans Affairs Medical Center, Atlanta, GA; 13 School of Medicine and Public Health, Emory University, Atlanta, GA, USA; 14 Geriatric Research, Education, and Clinical Center, Veterans Affairs Boston Healthcare System, Boston, MA; 15 Department of Biostatistics, Boston University School of Public Health, Boston, MA, USA

**Keywords:** Mega-cohort, Million Veteran Program, Veteran health care

## Abstract

**Aim:**

Million Veteran Program (MVP) is the largest ongoing mega-cohort biobank program in the US with 570,131 enrollees as of May 2017. The primary aim is to describe demographics, military service, and major diseases and comorbidities of the MVP cohort. Our secondary aim is to examine body mass index (BMI), a proxy for general health, among enrollees.

**Materials and Method:**

The study population consists of Veterans who actively use the Veterans Health Administration in the US. Data evaluated in this paper combine health information from multiple sources to provide the most comprehensive demographic profile and information on height and weight of MVP enrollees. A standardized cleaning algorithm was used to curate the demographic variables for each participant in MVP. For height and weight, we derived a final data point for each participant to evaluate BMI.

**Statistical Analysis Used:**

Multivariable logistic regression was used to compare the differences in BMI categories across enrollment years adjusting for gender, race, and age. *P* < 0.05 was considered statistically significant. All analyses were conducted using Statistical Analysis System 9.2.

**Results:**

The MVP cohort consists of 90.4% of males with an average age of 61.9 years (standard deviation [SD] = 13.9). MVP is the largest multiethnic biobank cohort within the Veteran population with 73.9% White, 19.0% Black, and 6.5% Hispanic. The most common self-reported disease was hypertension (62.6%) for males and depression (47.5%) for females. Mean BMI was 29.7 kg/m^2^ (SD = 5.8) with 38.2% obese and 42.3% overweight.

**Conclusions:**

Our findings suggest that demographic representation in MVP is similar to the Veterans Health Administration population and contrasts with the overall National Health and Nutrition Examination Survey US population. The prevalence of overweight and obese is high among US Veterans, and future studies will examine the role of BMI and disease risk in the Veteran population.

## INTRODUCTION

Million Veteran Program (MVP) is a mega-biobank cohort that was launched to establish a national, representative, and longitudinal study of Veterans that combines the data from survey instruments, electronic health records, genomics, and biospecimens. It is a noninterventional study that poses minimal risk to participants due to its observational design. Details on the design of MVP have been previously described.^[1]^ The objective of MVP is to understand how genetic characteristics, behaviors, military exposure, and environmental factors affect health. Ultimately by providing a framework for scientifically valid and clinically relevant precision medicine, MVP’s goal is to enhance the care of the Veteran population and beyond.

On August 1, 2016, MVP reached the half-million enrollment milestone, and as of May 2017, there were 570,131 enrollees. Demographic information collected from MVP surveys and the Department of Veterans Affairs (VA) electronic health records has been curated separately, but the usefulness of combining the two data sources for research has not been previously explored. In this paper, we report on the demographic composition of MVP participants and compare the demographic information using survey data and electronic health records to determine the best practice for characterizing the MVP cohort in future studies. We focus on body mass index (BMI), an important risk factor for health and disease that is influenced by both genetic and environmental determinants, to illustrate the phenotype data curation process and to examine how cross-sectional data may be effectively utilized for MVP research.

## MATERIALS AND METHODS

### Study population

The MVP VA Central Institutional Review Board (IRB) 10–02 protocol was approved in 2010 by the VA Central IRB, and study participant enrollment began in early 2011. The source population for the study is the 8.9 million users of the Veterans Health Administration (VHA). MVP is recruiting at approximately 50 VA facilities nationwide. Eligible candidates include registered users of the VHA at least 18 years of age with a valid mailing address and the ability to provide informed consent (~6.9 million Veterans). The target sample size for MVP is at least one million Veterans.

### Data sources

Self-reported demographics, family pedigree, health status, lifestyle habits, military experience, medical history, family history of specific illnesses, and physical features are collected in the MVP Baseline Survey.^[1]^ MVP’s waiver of consent and HIPAA for collection and use of the MVP Baseline Survey allow us to obtain data from surveys before and after a participant’s consent and enrollment. Supplemental data for missing self-reported demographic information were obtained from electronic health records in the VA corporate data warehouse.^[2,3]^ The National Health and Nutrition Examination Survey (NHANES) data for 2011–2014 MVP enrollment years were used to represent the demographic profile of the overall US population; NHANES data for 2015–2017 were not available.

A survey cleaning algorithm was created to scrub raw self-reported data from the MVP Baseline Survey. This multi-step process is broken down into components based on the question formats and response types. Criteria to establish rules for acceptable response values were determined by a panel of subject matter experts and statistical analysts. Values for each cleaned variable are shown in Supplemental Table 1. Electronic health record data supplemented where self-reported data were out of range or missing unless otherwise indicated.

### Demographic characteristics

Age was calculated from the electronic health records’ date of birth (DOB) to the MVP Baseline Survey completion date if available; otherwise, age was calculated to the enrollment date. If electronic health records’ DOB was missing, self-reported DOB was used. The acceptable age range was 18 years (minimum age requirement for military service) to 117 years (not being born before the year 1900). If no clear DOB was determined or was inconsistent across data sources, the participant’s age was considered missing.

Self-reported gender was categorized as selecting either male or female. If no response was indicated or a conflicting response was selected, the self-reported gender was considered missing/invalid and the most frequent gender recorded in the participant’s electronic health records was designated as the final gender.

The possible categories for ethnicity and race on the MVP Baseline Survey are presented in Supplemental Table 1. If conflicting ethnicity responses were selected on the MVP Baseline Survey, the most frequently reported ethnicity response in the electronic health records was used. To match electronic health records’ responses for race, we combined the following values into a single “Asian” category: “Chinese,” “Japanese,” “Asian Indian,” “Filipino,” and “Other Asian.” Selection of multiple races and military service eras on the survey was allowed, and “multiple” categories were considered valid.

Self-reported health conditions including cancers were determined by the responses to question 43 of the MVP Baseline Survey^[1]^ where all selections were considered valid. If no response to a demographic question was available from either survey or electronic health records, the response was coded to “missing.”

### Lifestyle and health factors

Alcohol usage and smoking status are not consistently available in the electronic health records but are asked on the MVP Baseline and Lifestyle Surveys. Using survey responses regarding beer, wine, and spirits, and drinks per day consumed, total ethanol was computed to derive drinks/day. Smoking status (“never,” “former,” or “current”) was derived from MVP Baseline and Lifestyle Surveys.

### Height and weight

Before combining heights and weights, we checked the reliability of electronic health records’ data by selecting a subset of participants with a baseline and electronic health records’ height or weight obtained 2 months of the baseline date. Pearson’s correlation coefficient and interclass correlation coefficient^[4]^ calculated to examine agreement among continuous data.

To determine final height and weight, we compared the Veteran’s electronic health records and self-reported values to ensure that the final value was consistent with the available data. Electronic health records’ data around the baseline/enrollment date were checked for outliers before comparing to self-reported data. Self-reported data close to the mean electronic health records’ data were We conducted descriptive analysis on sociodemographic considered acceptable. Self-reported data far from the characteristics, weight, and height to characterize the mean electronic health records’ data were removed and the electronic health records’ data were the final value. If self-reported data were not available, electronic health records’ data closest to the baseline/enrollment date were used. The details of height and weight cleaning algorithm can be found in Supplemental Figures 1 and 2.

Participants with low heights and a code for amputation were excluded from our analysis, but wheelchair and stretcher-bound participants were included. Within the electronic health records, we performed logic checks for height values and considered cancer diagnoses with weight as cancer treatment is strongly correlated with significant weight loss.^[5]^

### Body mass index

The final height and weight values were used to compute BMI, calculated as weight in kilograms divided by height in meters squared. BMI was classified into the following categories based on clinical guidelines:^[6]^ underweight (<18.5 kg/m^2^), normal weight (18.5–24.9 kg/ m^2^), overweight (25.0–29.9 kg/m^2^), Class I obese (30–34.9 kg/m^2^), Class II obese (35–39.9 kg/m^2^), or Class III obese (≥40 kg/m^2^).

### Data analysis

We conducted descriptive analysis on sociodemographic characteristics, weight, and height to characterize the MVP cohort. For BMI analyses, we used standard Statistical Analysis System (SAS) procedures to determine average BMI in MVP. A similar approach was taken for VHA-wide data using electronic health records. For NHANES, we followed established survey methods and analytic guidelines. Multivariable logistic regression was used to compare the differences in BMI categories across enrollment years adjusting for gender, race, and age. A *P* < 0.05 was considered statistically significant. All analyses were conducted using SAS 9.2 (SAS Institute Inc., Cary, NC, USA).

## RESULTS

### Demographics

As of May 24, 2017, there were 570,131 Veterans enrolled in MVP and 3779 of these individuals have withdrawn. A total of 362,360 (63.3%) enrollees returned a MVP Baseline Survey with 262,326 (72.6%) completing the survey by enrollment date. Of the remaining enrollees, 75% returned the completed survey within 50 days of enrollment. The mean interval between enrollment and survey return date was 41 days.

Characteristics of the MVP cohort are presented in Tables 1 and 2. The MVP population is primarily male (90.4%) with a mean age of 61.9 (13.9) years. The mean age for female participants was 50.7 (13.5) years. Compared to the general nondeceased VHA population (*n* = 11,494,756), MVP participants are younger and have a higher proportion of Blacks.

**Figure 1:**
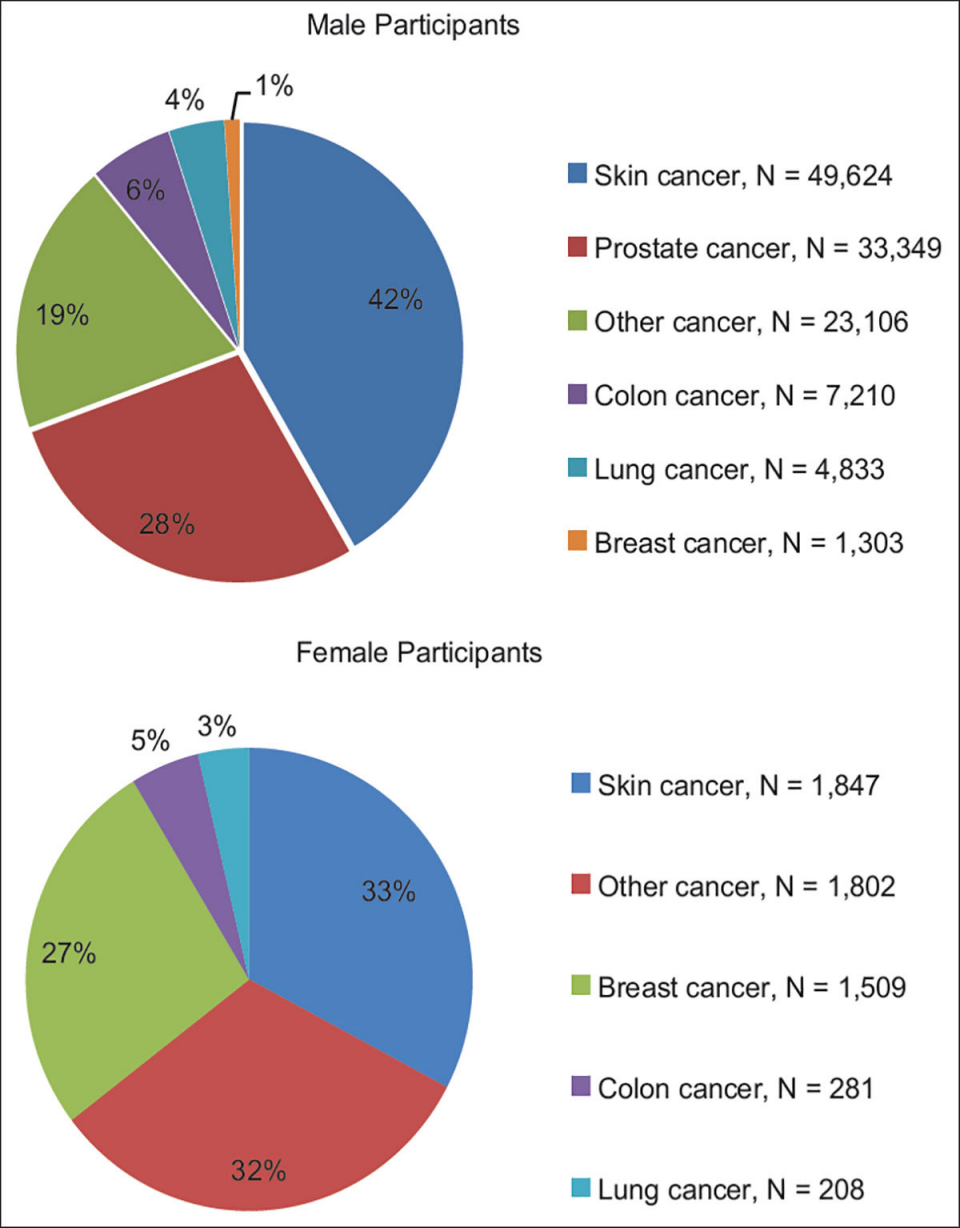
Self-reported cancers among Million Veteran Program participants

The most prevalent self-reported disease in MVP was hypertension (62.6%). When stratified by gender, hypertension remained the most prevalent disease among males (64.6%) and depression (47.5%) was the most prevalent disease among females [Table 3]. A total of 125,228 participants reported having had cancer; the most common self-reported cancer for males and females after skin cancer was prostate cancer (*n* = 33,349) and other cancer (*n* = 1847), respectively [Figure 1].

### Body mass index

Less than 1% of self-reported height values were invalid compared to 1.5% of electronic health record-reported height measurements. Self-reported weight also had fewer invalid responses compared to electronic health record-reported weight within a 3-year period of enrollment (1.6% vs. 4.0%) (data not shown). Self-reported height (*R*^2^ = 0.89) and weight (*R*^2^ = 0.96) were highly correlated with electronic health record measurements obtained within 2 months of baseline date. Thus, electronic health records’ data were used to supplement invalid/missing self-reported data to analyze BMI for all MVP enrollees.

A total of 548,197 MVP enrollees were included in the BMI analysis. 21,934 (3.8%) participants were excluded due to invalid/missing height and/or weight. In the final cohort, height and weight were supplemented with 194,248 and 220,517 electronic health records’ observations, respectively. The mean BMI was 29.7 kg/m^2^ (5.8) for MVP enrollees.

Mean BMI was similar in males (29.7 kg/m^2^) and females (30.0 kg/m^2^). While males were borderline obese regardless of race, Black females had a higher BMI than their White counterparts (31.1 vs. 29.6 kg/m^2^, respectively). The majority of Veterans, regardless of gender and race, have a BMI ≥30.0 kg/m^2^. At the extreme, 5.1% of White males and 6.0% of Black males had BMI ≥40 kg/m^2^, and among females, 7.8% of Whites and 9.0% of Blacks had BMI ≥40 kg/m^2^ [Table 4].

### Body mass index trends

**Figure 2:**
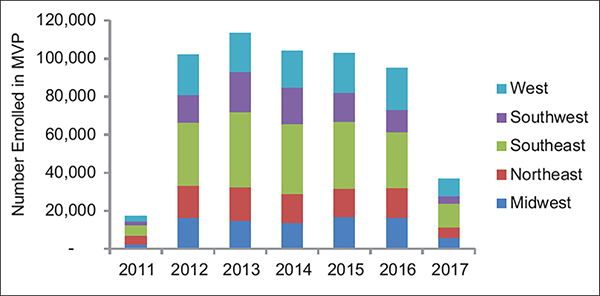
Frequency of Million Veteran Program enrollees by US enrollment region through May 2017

Enrollment remained steady with at least 100,000 enrollees each year between 2012 and 2015 [Table 5]. The Southeast region of the US saw the highest enrollments across all years [Figure 2]. The prevalence of borderline Class I obese was steady across enrollment years for males and females, with the BMI ranging from 29.2 to 31.5 kg/m^2^ between January 2011 and May 2017. The Midwest had the highest BMI followed by the southwest, southeast, west, and northeast. The Midwest showed the greatest overall upward trend in BMI across enrollment years (data not shown).

**Figure 3:**
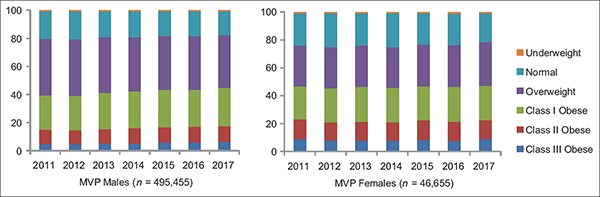
Trends in body mass index by enrollment year (2011–2017) across weight categories for males and females

The prevalence of obesity increased in the MVP population over time [Figure 3]. When adjusted for gender, age, and race, the observed increase in obesity was statistically significant (*P* < 0.001). The odds ratio (OR) (95% confidence interval [CI]) of being obese in 2016 and 2017 compared to 2011 was 1.13 (1.09, 1.17) and 1.19 (1.15, 1.24), respectively. There was a significant decrease in the prevalence of overweight individuals from 2011 to 2017 after adjusting for gender, age, and race (*P* < 0.001). The OR (95% CI) of being overweight in 2016 and 2017 compared to 2011 was 0.93 (0.90, 0.96) and 0.91 (0.87, 0.94), respectively. There was no significant change in normal weight participants enrolling in MVP from 2011 to 2017. No trend was observed in the underweight group across enrollment years.

## DISCUSSION

Fewer than 20% of male Veterans and 30% of female Veterans had a normal BMI at the time of MVP enrollment. The prevalence of overweight BMI among MVP enrollees decreased with each successive year, while the prevalence of obesity increased. We found that combining health information from multiple data sources provided the most comprehensive demographic profile of MVP enrollees.

The MVP population is representative of the overall VHA population with regard to mean age and gender distribution. Ethnicity distributions were difficult to compare because >30% of electronic health records’ ethnicity data were missing, whereas only 1.6% of ethnicity information was missing for MVP participants. We observed differences between the MVP and NHANES populations. MVP participants are older (mean age: 61.9 [13.9] vs. 46.9 [0.5] years) and are more likely to be seeking health care compared to the general US population. When we restricted NHANES to those ≥45 years, the mean age of the subgroup was 50.5 (0.2) years. The distribution of males and females was comparable in NHANES, whereas the MVP (and VHA) population consists primarily of male Veterans. Distribution of ethnicity was similar across MVP and the NHANES population ≥45 years with 90% classified as non-Hispanic. Whites were the majority racial group; however, Blacks and Hispanics had higher relative representation in MVP compared to NHANES. The current enrollment of over 100,000 Blacks and over 35,000 Hispanics in MVP provides the largest research cohort in the US to study the genetic and environmental determinants of disease.

### Million Veteran Program demographic data and self-reported health conditions

Combining multiple data sources optimizes the amount of data available to characterize the demographic profile of MVP participants. Our combined demographic data also provide data mappings and standardization that allow researchers to use a single measure for a demographic variable. We found that <5% of responses to DOB, race, and sex on the MVP Baseline Survey disagreed with the data in the electronic health records (data not shown). When we augmented self-reported data with electronic health records’ data, we were able to obtain at least one demographic variable for every individual participating in MVP, and thus, no enrollees were excluded. Furthermore, the MVP Baseline Survey lists 75 diseases and conditions for self-report [Table 3 and Figure 1], which will provide an opportunity to investigate these conditions within a multiethnic cohort that is representative of the wider VHA population.

### Analysis of body mass index

Consistent with the recent NHANES findings, the prevalence of obesity among females was higher than among males in MVP.^[7]^ However, there was a higher prevalence of obesity in both males (42.0%) and females (46.2%) compared to the NHANES US population where the prevalence of obesity is 35.0% and 40.4% for males and females, respectively.^[8]^ The higher prevalence of obesity in females persisted across gender and race subgroups. Black females have a significantly higher prevalence of overweight and obesity than White females, a finding that is supported by the previous studies that have examined gender and racial differences in BMI.^[9–12]^

Previous studies have examined overweight and obese prevalence in VHA users vs VHA nonusers and found that users are more overweight and obese than non-VHA users.^[13,14]^ Our data support the claim that VHA users in MVP are more overweight and obese than the general population. Veterans who utilize the VHA as their primary source for healthcare have more adverse health conditions, lower income, and lower education compared to non-VHA Veterans.^[15]^ The higher prevalence of obesity in MVP may be due to these factors, which have been associated with higher rates of obesity.^[9]^ Among active-duty military personnel, the prevalence of overweight and obesity was 61%,^[16,17]^ and given that increasing age is significantly associated with obesity among civilian and active duty military personnel, the observed prevalence of overweight and obesity (80.5%) in our older MVP population is not surprising.^[16,18]^ The higher rates of obesity may be a result of Veterans aging and gaining small amounts of weight over many years.^[19,20]^

Overall trends in US adult obesity and BMI showed a steady increase from 1980 to 2000 according to the NHANES, and the prevalence of obesity Types I, II, and III has remained unchanged since 2003.^[21–23]^ Our data suggest that the prevalence of overweight decreased across enrollment years while the prevalence of obesity increased. Obesity in the US is associated with an increase in morbidity and mortality that imposes considerable burdens on individuals’ public health,^[24,25]^ particularly in older adults,^[26,27]^ as well as to the healthcare system.^[28]^ The similarities with the NHANES data in regard to overweight and obesity suggest that studies in MVP may be generalizable to the US population.

### Limitations and strengths

We acknowledge the limitation that using BMI as an estimate of body fat may misclassify Veterans as overweight or even obese due to excess lean body mass.^[12,14,28]^ The use of our BMI variable in the future MVP studies should consider supplementing BMI with newer measures such as waist-to-height ratio or lean mass.^[29,30]^ A study using NHANES showed that between 1988–1994 and 2005–2006, there was an increase in mean waist circumference within all BMI categories, race, gender, and education.^[31]^ Other studies have suggested that using a waist-to-height ratio is a better indicator of risk factors associated with adiposity.^[32]^ Our study is limited to cross-sectional BMI data as MVP surveys have only been administered once. A single BMI measurement for each participant leaves the data susceptible to misclassification as small fluctuations in weight can result in large changes in BMI. Therefore, it is important to examine how the distribution of repeated BMI measures changes over time. Self-reported height and weight may be biased; weight is often under-estimated and height is often overestimated, but the degree of misreporting differs slightly between males and females.^[33,34]^ To minimize this potential bias, we used patient electronic health records to validate the self-reported height and weight.

The MVP surveys and electronic health records also limit gender to male and female assignments. In recent years, there has been a shift to include multiple categories of gender, including transgender and nonbinary gender identity, which will be considered in the future MVP surveys.

Data management challenges arise due to the high volume and variability in the data collected for a mega-cohort study. A Veteran can have multiple measurements in the electronic health records for a single variable that may be inconsistent over time. Similarly, there can be inconsistencies among different data sources for a single variable. To address the volume and variability within the MVP data, we used self-reported information to compare and validate medical history data in the electronic health records. This study aims to set the foundation of managing and preparing health care data from multiple sources for research by describing our cleaning and curation methods. Additional strengths of this study are the large sample size, racial and ethnic diversity, and broad geographical representation of Veterans across the US. Our MVP core demographic data provide the most complete data source, because it is validated using multiple data sources and enriched with self-reported lifestyle behavior data that are not readily available in the electronic health records.

The similarities in trends between MVP and NHANES suggest that lessons learned in MVP may generalize. By combining medical record and questionnaire data on height and weight as shown in this study, MVP establishes itself as a valuable resource for determining ways to improve the health of, and the delivery of health care to, US Veterans and the general population. Future investigation will take advantage of the longitudinal electronic health records and include population-based studies to examine factors of weight gain and obesity in Veterans with the aim of reducing the health outcome and economic cost of the obesity epidemic. MVP presents a unique opportunity to advance the fields of population phenomics and genomics and develop precision medicine approaches to optimize care and quality of life for Veterans, the US population, and beyond.

## Supplementary Material

Supplemental Figure 2

Supplemental Figure 1

Supplemental Table 1 and 2

## Figures and Tables

**Table 1:** Baseline characteristics of the MVP cohort

Characteristic	MVP		VHA		NHANES	
	Males	Females	Males	Females	Males	Females
	(*n*=515,399)	(*n*=49,039)	(*n*=10,668,917)	(*n*=788,171)	(*n*=110,877,951)	(*n*=119,568,087)

Demographics						
Age (years), mean±SD	63.0±13.4	50.7±13.5	67.8±18.6	52.3±17.2	62.0±0.1	50.3±0.1
Gender (%)						
Male	-	-	-	-	-	-
Female	-	-	-	-	-	-
Missing	-	-	-	-	-	-
Ethnicity (%)						
Non-Hispanic	92.1	91.3	63.5	67.6	90.2	86.8
Hispanic	6.5	7.1	4.2	5.4	9.8	13.2
Missing	1.4	1.6	32.4	27.0		
Race (%)						
White	75.7	63.2	53.7	47.3	74.6	66.9
Black	18.2	29.0	10.3	21.4	9.2	12.2
Asian	1.0	1.5	0.8	1.3	4.2	5.4
American Indian/Alaska	1.0	1.7	0.7	1.2	-*	-*
Native (%)						
Native Hawaiian/other pacific	0.5	0.6	0.7	1.0	-*	-*
Islander (%)						
Other	1.3	1.6	-	-	12.0	15.5
Multiple	2.5	4.8	0.5	1.0	-	-
Missing	2.3	2.6	34.4	28.9		-
Service era (%)^†^						
September 2001 or later	10.3	25.6	9.4	16.9	-	-
August 1990 to August 2001	20.2	52.0	23.9	58.6	-	-
May 1975 to July 1990	21.9	37.4	11.7	20.4	-	-
August 1964 to April 1975	52.3	17.0	35.3	10.4	-	-
February 1955 to July 1964	11.6	2.8	5.6	1.5	-	-
July 1950 to January 1955	7.8	1.4	9.2	1.9	-	-
January 1947 to June 1950	1.1	0.1	0.5	0.1	-	-
December 1941 to December 1946	3.4	0.9	12.3	4.5	-	-
November 1941 or earlier	0.1	0.0	0.2	0.0	-	-
Multiple	20.9	30.3	9.8	17.2	-	-
Missing	0.3	0.9	1.8	3.0	-	-
Health history						
BMI (kg/m^2^), mean±SD	29.7±5.8	30.1±6.7	29.6±6.0	29.9±6.7	28.9±0.3	29.6±0.3
Height (inches)	69.9 (2.8)	64.8 (2.8)	69.6 (2.9)	64.7 (2.9)	68.8 (0.2)	63.7(0.1)
Weight (lbs)	206.6 (43.6)	179.4(42.0)	202.7 (44.2)	175.9 (41.8)	195.1 (2.4)	171.0 (2.1)
Systolic blood pressure (mmHg), mean±SD	131.7±17.0	126.1±16.4	132.0±16.9	125.4±16.9	128.8±1.0	121.1±0.8
Diastolic blood pressure (mmHg), mean±SD	77.1±10.9	76.4±10.4	76.5±10.9	75.9±10.5	71.0±0.7	70.9±0.5
Smoking (%)						
Never	24.9	46.4	-^‡^	-^‡^	42.0	60.1
Former	57.4	34.6	-	-	40.8	21.5
Current	17.7	19.1	-	-	17.2	18.4
Alcohol (%)						
Never	8.0	12.6	-^‡^	-^‡^	32.9	36.4
0-1 drinks/day	31.3	38.1	-	-	24.1	30.5
1-2 drinks/day	9.0	6.0	-	-	20.7	19.8
2-3 drinks/day	3.9	2.0	-	-	9.6	6.9
3-4 drinks/day	2.7	0.8	-	-	4.4	2.7
4+ drinks/day	3.4	1.2	-	-	8.3	3.7

* Not a race category m NHANES

† Percents will add up to >100% due to multiple answers being allowed

‡ Data not available m electronic health records. BMI: Body mass index, NHANES: National Health and Nutrition Examination Survey, MVP: Million Veteran Program, VHA: Veterans Health Administration

**Table 2:** Health and lifestyle characteristics of the MVP cohort

Characteristic	MVP			VHA	NHANES
	Males	Females	Total	(*n*=11,494,756)	(*n*=230,446,039)
	(*n*=515,399)	(*n*=49,039)	(*n*=570,131)		

BMI (kg/m^2^), mean±SD	29.7±5.8	30.1±6.7	29.7±5.8	29.6±6.0	28.9±0.1
Height (inches), mean±SD	69.9±2.8	64.8±2.8	69.5±3.2	69.3±3.2	66.3±0.1
Weight (lbs), mean±SD	206.6±43.6	179.4±42.0	204.3±44.1	200.7±44.5	181.4±0.8
Systolic blood pressure (mmHg), mean±SD	131.7±17.0*	126.1±16.4*	131.2±17.1*	131.5 (17.4)	121.8 (0.3)
Diastolic blood pressure (mmHg), mean±SD	77.1±10.9*	76.4±10.4*	77.1±10.8*	76.5 (10.9)	70.7 (0.3)
Smoking (%)					
Never	24.9	46.4	26.5	-^†^	56.4
Former	57.4	34.6	55.6	-	23.8
Current	17.7	19.1	17.8	-	19.8
Alcohol (%)					
Never	8.0	12.6	8.4	-^†^	32.5
0-1 drinks/day	31.3	38.1	31.8	-	23.4
1-2 drinks/day	9.0	6.0	8.8	-	19.4
2-3 drinks/day	3.9	2.0	3.8	-	9.5
3-4 drinks/day	2.7	0.8	2.5	-	5.1
4+ drinks/day	3.4	1.2	3.2		10.0

* Blood pressure as presented was not asked on MVP surveys; data, if available, is from electronic health records

† Smoking status and alcohol intake as presented categories are not available in electronic health records. BMI: Body mass index, NHANES: National Health and Nutrition Examination Survey, MVP: Million Veteran Program, VHA: Veterans Health Administration, SD: Standard deviation

**Table 3:** Self-reported health conditions among participants who completed the MVP Baseline Survey (*n*=362,260)

Disease categories	Male, *n* (%)	Female, *n* (%)

Heart and circulatory		
High blood pressure (hypertension)	214,716* (64.6)	12,006* (40.9)
Stroke	24,097 (7.2)	1,072 (3.7)
TIA	16,199(4.9)	867 (3.0)
Heart attack	43,454(13.1)	987 (3.4)
Coronaiy artery/coronary heart disease (includes angina)	57,040 (17.2)	1,323 (4.5)
Peripheral vascular disease	18,043 (5.4)	614(2.1)
High cholesterol	190,974* (57.4)	12,295* (41.9)
Pulmonary embolism or deep vein thrombosis	12,631 (3.8)	933 (3.2)
Congestive heart failure	23,202 (7.0)	770 (2.6)
Other circulatory system problem	31,310(9.4)	1,732 (5.9)
Musculoskeletal		
Osteoarthritis	48,627 (14.6)	7,339* (25.0)
Rheumatoid arthritis	31,030 (9.3)	2,214 (7.5)
Other arthritis	78,067* (23.5)	6,301 (21.5)
Gout	37,016(11.1)	761 (2.6)
Osteoporosis	11,714 (3.5)	3,320(11.3)
Other skeletal/muscular problem	65,470 (19.7)	8,339* (28.4)
Mental health		
Anxiety reaction/panic disorder	52,449 (15.8)	8,910* (30.4)
ADHD	10,774 (3.2)	1,330(4.5)
Bipolar disorder	12,496 (3.8)	2,451 (8.4)
PTSD	60,714(18.3)	7,643* (26.0)
Depression	89,116* (26.8)	13,941* (47.5)
Eating disorder	9,655 (2.9)	1,116(3.8)
Personality disorder	10,290 (3.1)	1,344(4.6)
Schizophrenia	6,261 (1.9)	494(1.7)
Social phobia	9,887 (3.0)	1,027 (3.5)
Other mental health disorder	13,276 (4.0)	1,744 (5.9)
Hearing/vision		
Cataracts	89,275* (26.8)	4,451 (15.2)
Glaucoma	26,441 (8.0)	1,306(4.5)
Macular degeneration	18,336 (5.5)	911 (3.1)
Blindness, all causes	8,457 (2.5)	398(1.4)
Tinnitus	113,841* (34.2)	5,882 (20.0)
Heaiing loss	106,497* (32.0)	3,023 (10.3)
Infectious		
Tuberculosis	7,141 (2.1)	619(2.1)
Hepatitis C	17,395 (5.2)	768 (2.6)
HIV/AIDS	4,567 (1.4)	175 (0.6)
Other infectious diseases	8,758 (2.6)	1,013 (3.5)
Kidney		
Kidney disease (no dialysis)	19,030 (5.7)	836 (2.8)
Kidney disease (with dialysis)	3,342 (1.0)	118(0.4)
Acute kidney disease (no dialysis)	5,465 (1.6)	219(0.7)
Digestive system		
Acid reflux/GERD	109,507* (32.9)	10,954* (37.3)
Peptic ulcers	12,826 (3.9)	1,144 (3.9)
Bowel obstruction	9,372 (2.8)	726 (2.5)
Colon polyps	74,124 (22.3)	3,814(13.0)
IBS	14,351 (4.3)	3,932(13.4)
Ulcerative colitis	5,072 (1.5)	489(1.7)
Crohn’s disease	3,033 (0.9)	265 (0.9)
Celiac disease/sprue	1,810(0.5)	242 (0.8)
Other digestive system disorder	18,423 (5.5)	2,582 (8.8)
Nervous system		
Migraine headaches	27,513 (8.3)	8,692* (29.6)
Other headaches	33,637(10.1)	5,079 (17.3)
Memoiy loss or impairment	34,970 (10.5)	2,802 (9.6)
Dementia	5,233 (1.6)	191 (0.7)
Concussion or loss of consciousness	27,272 (8.2)	2,903 (9.9)
Traumatic brain injury	11,634 (3.5)	1,133 (3.9)
Spinal cord injury or impairment	24,067 (7.2)	1,387(4.7)
Epilepsy/seizure	7,508 (2.3)	836 (2.8)
Parkinson’s disease	4,797 (1.4)	135 (0.5)
ALS/Lou Gehrig's disease	1,224 (0.4)	55 (0.2)
Multiple sclerosis	2,446 (0.7)	495 (1.7)
Other nervous system problem	20,298 (6.1)	2,154 (7.3)
Other conditions		
Asthma	27,515 (8.3)	5,158 (17.6)
Chronic lung disease	38,104(11.5)	2,799 (9.5)
Diabetes	92,241* (27.7)	4,613 (15.7)
Enlarged prostate	68,980 (20.7)	-
Liver condition	10,879 (3.3)	656 (2.2)
Skin condition	35,334 (10.6)	3,726 (12.7)
Sleep apnea	92,166* (27.7)	5.380 (18.3)
Thyroid problems	29,544 (8.9)	6,372* (21.7)
Other disease/disorder	12,836 (3.9)	2,521 (8.6)

* Top ten most reported within each gender group. TLA: Transient ischemic attack, IBS: Irritable bowel syndrome, GERD: Gastroesophageal reflux disease, ALS: Amyotrophic lateral sclerosis, PTSD: Posttraumatic stress disorder

**Table 4:** Body mass index characteristics by gender and race using MVP core demographic data

BMI (kg/m^2^) characteristic	Overall (*n*=545,940) (%)	Male (*n*=495,455)			Female (*n*=46,655)		
		White (11=368,124) (%)	Black (*n*=87,237) (%)	Other (*n*=40,094) (%)	White (*n*=28,358) (%)	Black (*n*=12,832) (%)	Other (*n*=5,465) (%)

Mean±SD	29.7±5.8	29.6±5.7	29.6±5.7	29.9±5.8	29.6±6.8	31.1±6.4	29.7±6.4
Range	11.5-79.1	11.5-79.1	11.6-77	15.0-73.8	12.0-77.5	13.4-74.1	15.2-65.5
Underweight (< 18.5)	0.6	0.5	0.9	0.5	1.3	0.6	0.9
Normal (18.5-24.9)	18.8	18.5	18.9	17.1	26.0	15.5	24.5
Overweight (25.0-29.9)	38.2	39.7	35.8	39.2	29.5	30.3	30.5
Class I obese (30.0-34.9)	25.8	25.7	26.9	26.3	23.0	28.4	24.6
Class H obese (35.0-39.9)	11.0	10.5	11.6	11.4	12.6	16.2	12.5
Class III obese (>40)	5.5	5.1	6.0	5.5	7.8	9.0	7.0

BMI: Body mass index, SD: Standard deviation

**Table 5:** MVP enrollment by year

Enrollment year	2011	2012	2013	2014	2015	2016	2017*

Number enrolled (%)	17,115	10,2070	113,544	103,946	102,725	95,146	35,585

* Data only through May 2017
