# Supplementary figures and images for: Baseline Characterization and Annual Trends of Body Mass Index for a Mega-Biobank Cohort of US Veterans 2011–2017
Source: J Health Res Rev Dev Ctries. Author manuscript; Available in PMC 2020 Oct 27. (PMC7590919)

Supplemental Figure 2. Weight Cleaning Algorithm

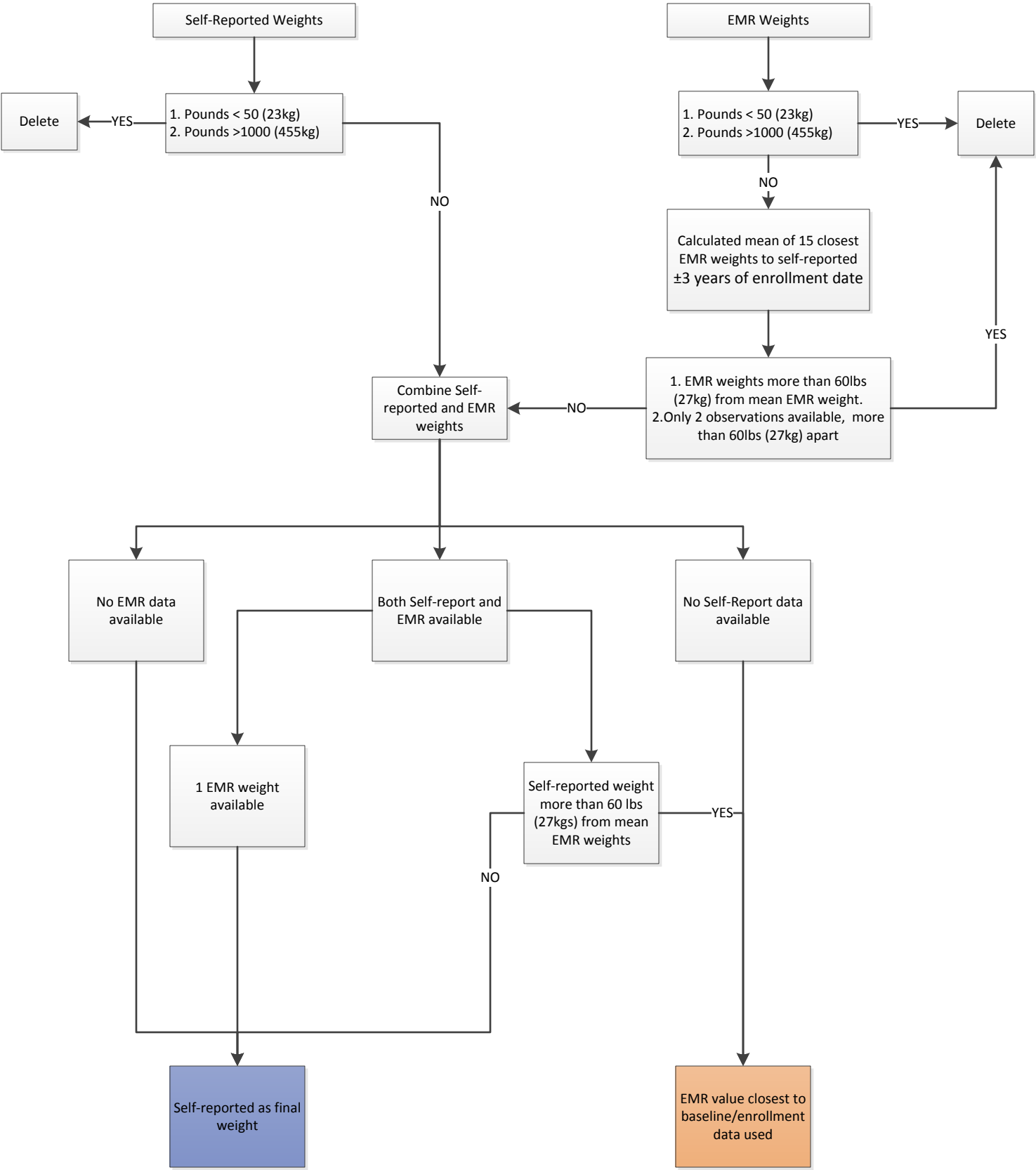

Supplement: Supplemental Figure 2 [file NIHMS1625200-supplement-Supplemental_Figure_2.pdf]

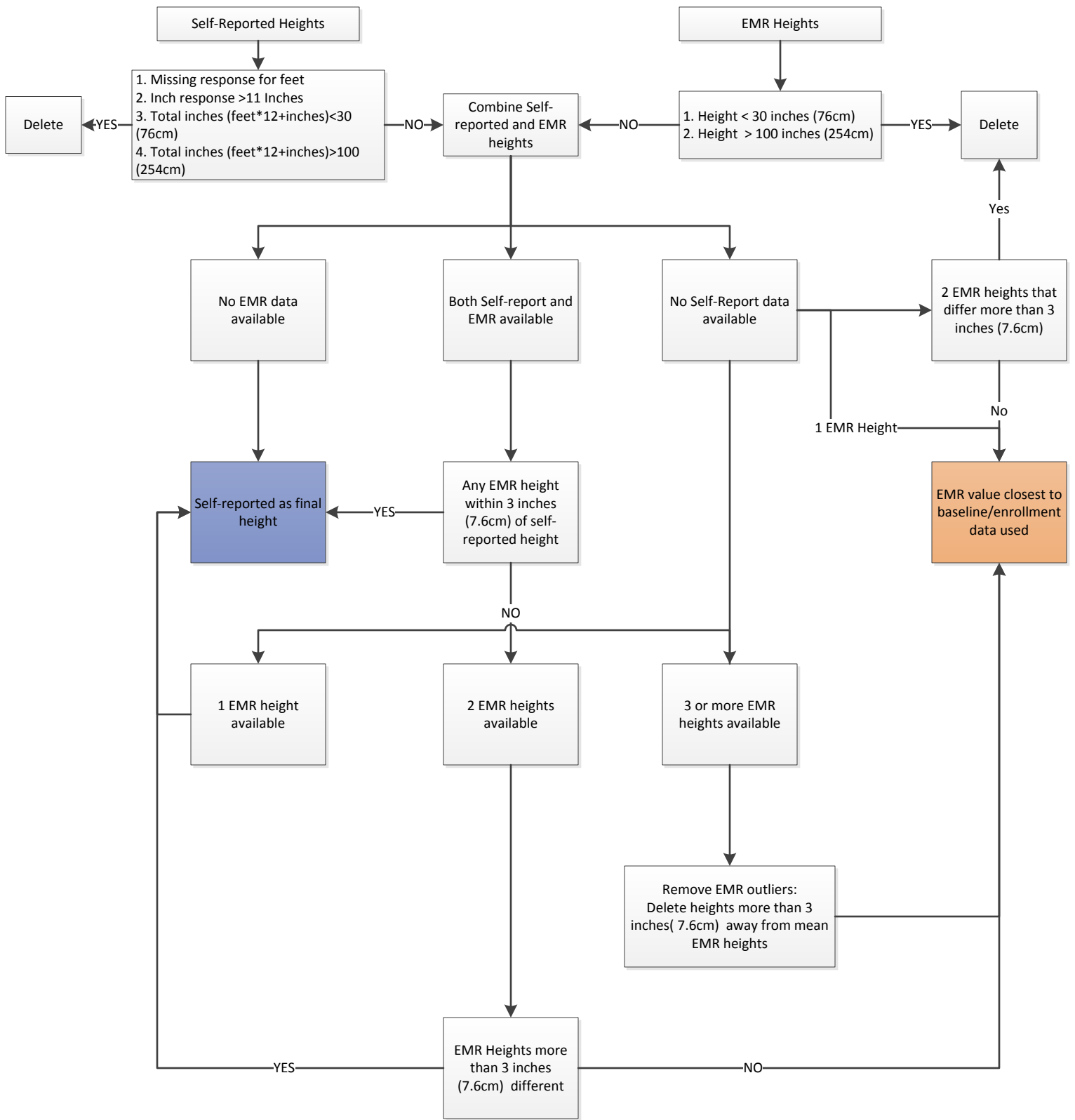

Supplement: Supplemental Figure 1 [file NIHMS1625200-supplement-Supplemental_Figure_1.pdf]
