# Supplementary material for: Baseline Characterization and Annual Trends of Body Mass Index for a Mega-Biobank Cohort of US Veterans 2011–2017
Source: J Health Res Rev Dev Ctries. Author manuscript; Available in PMC 2020 Oct 27. (PMC7590919)
Supplement: Supplemental Table 1 and 2 [file NIHMS1625200-supplement-Supplemental_Table_1_and_2.pdf]

Supplemental Table 1: Possible values for each variable by source		
	**MVP survey**	**EHR**
Age	DOB	DOB
Gender	Male	Male
	Female	Female
Ethnicity	No, Not Spanish, Hispanic, Latino	Hispanic or Latino
	Yes, Mexican, Mexican American, Chicano	Not Hispanic or Latino
	Yes, Puerto Rican	Unknown or Null
	Yes, Cuban	Declined to answer
	Yes, Other Spanish, Hispanic, Latino	
Race*	White	White
	Black/African-American	Black or African-American
	American Indian/Alaska Native	Asian
	Chinese	American-Indian or Alaska Native
	Japanese	Native Hawaiian or Other Pacific Islander
	Asian Indian	Multiple
	Other Asian	Unknown or null
	Filipino	
	Pacific Islander	
	Other	
Period of service*	September 2001 or later	OEF/OIF
	August 1990 to August 2001	AIR force-active duty
	May 1975 to July 1990	Army-active duty
	August 1964 to April 1975	CAV/NPS
	February 1955 to July 1964	CHAMPVA-spouse, child
	July 1950 to January 1955	Coast guard-active duty
	January 1947 to June 1950	Czechoslovakia/Poland SVC
	December 1941 to December 1946	Donors (nonvet)
	November 1941 or earlier	Humanitarian (nonvet)
		Inactive duty
		Job corps/peace corps
		Korean
		Medical remedial enlist
		Merchant marine
		Merchant marine-WWII
		Merchant marines
		Merchant seamen-USPHS
		Merchant seamen-USPHS
		Multiple
		Navy, marine-active duty
		Observation/examination
		Office of workers comp.
		Operation desert shield
		Other federal-dependent
		Other nonveterans
		Other or none
		Other reimburs (nonvet)
		Other USPHS beneficiaries
		Persian gulf war
		Post-Korean

Contd...

Supplemental Table 1: Contd...		
	MVP survey	EHR
		Post-Vietnam
		Pre-Korean
		Railroad retirement
		Retired, uniformed forces
		Spanish American
		Special studies (nonvet)
		Tricare
		Vietnam era
		World war I
		World war II
Height	Feet and inches	Inches
Weight	Pounds	Pounds

*Selections as they appear on the MVP baseline survey. Veterans may select all that apply. MVP: Million Veteran Program, EHR: Electronic health record, WWII: World War II, SVC: Service, USPHS: United States Public Health Service, OEF: Operation Enduring Freedom, OIF: Operation Iraqi Freedom, NPS: National Park Service, DOB: Date of birth

Supplemental Table 2: Comparison of demographic data between sources

	MVP Baseline Survey vs. EHR				Mismatch among people who have data in both sources	
	570,131 w/baseline MVP survey (*n*=361,393)		EHR (*n*=361,393)			
DOB						
Missing	8,631	2.39%	7,720	2.14%	5,852/319,037	1.82%
Invalid	95	0.03%	3	0.00%		
Gender						
Male	319,750	88.99%	325,788	90.15%	154/315,602	0.05%
Female	29,199	8.12%	27,909	7.72%	582/28,080	2.07%
Unknown or Missing	10,200	2.89%	7,696	2.13%	-	-
Race						
White	289,871	80.21%	273,547	75.69%	10,922/320,896	3.40%
Black	50,662	14.02%	48,201	13.34%	3,034/320,896	0.95%
Asian†	4,148	1.15%	2,649	0.73%	1,399/320,896	0.44%
Native Hawaiian or Other Pacific Islander	803	0.22%	3,178	0.88%	2,995/320,896	0.93%
American Indian or Alaska Native	13,363	3.72%	2,938	0.81%	10,841/320,896	3.38%
Multiple*	14,623	4.05%	2,431	0.67%	-	-
Other	11,086	3.07%	-	-	-	-
Unknown or Null‡	8,303	2.30%	33,390	9.24%	-	-
Height	69.6 (3.2)	-	69.5 (3.1)	-	-	0.7%
Weight	201.3 (42.8)	-	202.5 (42.5)	-	-	3.9%

*Using survey data from May 2017. †Asian includes the following MVP Baseline Survey categories: Chinese, Japanese, Asian Indian, Other Asian, Filipino ‡"Declined to Answer" is grouped under "Unknown or Null"

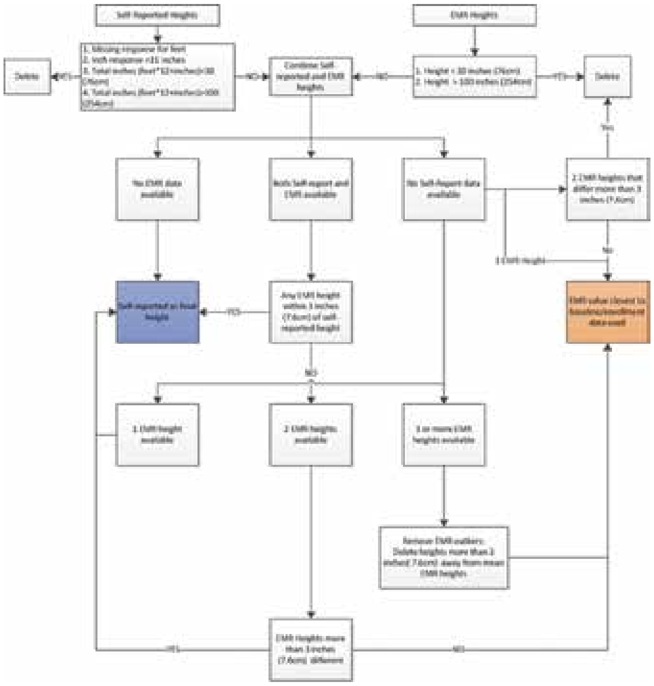

Supplemental Figure 1: Height cleaning algorithm

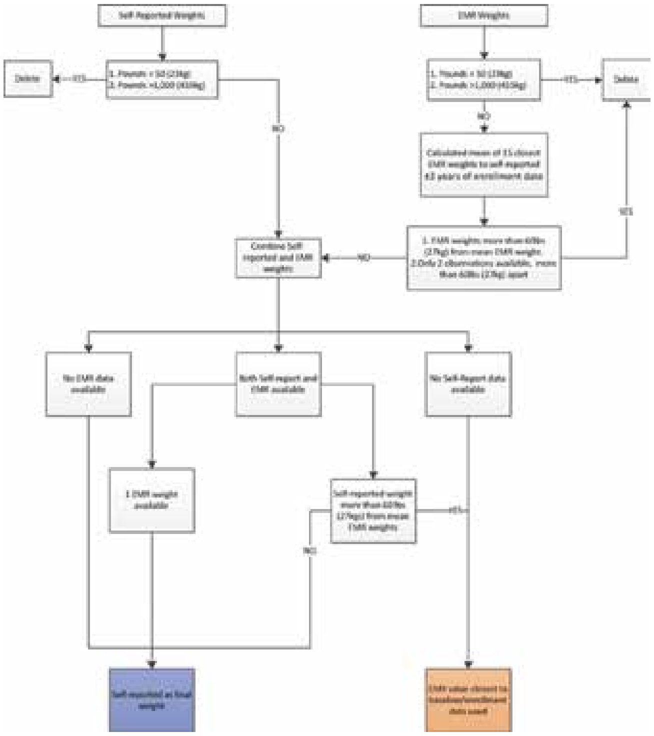

Supplemental Figure 2: Weight cleaning algorithm
